# Treatment options for small cell lung cancer – do we have more choice?

**DOI:** 10.1038/sj.bjc.6605527

**Published:** 2010-01-26

**Authors:** M Puglisi, S Dolly, A Faria, J S Myerson, S Popat, M E R O'Brien

**Affiliations:** 1 Department of Medicine, The Royal Marsden NHS Foundation Trust, Sutton, Surrey, UK

**Keywords:** small cell lung cancer, chemotherapy, relapsed SCLC, treatment

## Abstract

Small cell lung cancer (SCLC) is a significant health problem worldwide because of its high propensity for relapse. This review discusses existing and future therapies for the treatment of SCLC.

Each year approximately 1.4 million people worldwide are diagnosed with lung cancer – 12% of all new cases of cancer. Small cell lung cancer (SCLC) now represents only 13% of all newly diagnosed lung cancers. The SEER database indicates that the annual incidence of SCLC has been decreasing over the past 30 years (Field and Duffy, 2008), More than 90% of patients with SCLC are current or past smokers, and the risk is related to the duration and intensity of the smoking (Brownson *et al*, 1992). The median age at diagnosis in developed countries is approximately 68 years, and as many as 31% of patients may be aged 70 years or older (Yau *et al*, 2006).

## Staging

The SCLC is an aggressive disease associated with early loco-regional and distant metastases and paraneoplastic syndromes (Lally *et al*, 2007). It has a different staging system to that of other solid tumours (Chua *et al*, 2004). The Veterans Affairs Administration Lung Cancer Study Group (VALG) two-stage classification system introduced in 1957 the terms limited disease (LD) and extensive disease (ED) (Felip *et al*, 2005). Thirty years later, the consensus report of the International Association of Lung Cancer (IASLC) modified the VALG classification (Stahel *et al*, 1989). They recommended including patients with ‘ipsilateral and contralateral, hilar, mediastinal and supraclavicular nodes’ as LD. They also included patients with ipsilateral pleural effusion regardless of the cytology in the limited subgroup.

A Japanese series reported the comparison of a group of patients who received definitive thoracic radiotherapy (TRT) in which the pleural effusion had disappeared after induction chemotherapy, with a second group who did not receive TRT despite response and a third group with a pleural effusion that did not respond to chemotherapy. Long-term survival was achieved in those patients who successfully underwent chemoradiotherapy (Niho *et al*, 2008).

An ongoing retrospective survival analysis of data from >8000 SCLC patients in the IASLC database, many of whom were treated surgically, showed that survival of patients with LD and effusion is intermediate between those of patients with LD without effusion and patients with ED. The data suggest that clinical TNM staging can identify subgroups of patients with distinct prognoses within the conventional definition of LD and that all effusion should be cytologically proven (Shepherd *et al*, 2007). Such findings have led to the new recommendations for lung cancer staging based on the AJCC/UICC TNM seventh edition that are likely to supersede the VALG system in future clinical trials of SCLC (Detterbeck *et al*, 2009).

## Prognostic factors

Disease stage remains the most powerful prognostic factor for SCLC. The Manchester scoring system is frequently used in the clinic as an indicator of prognosis in SCLC. It is calculated from a number of physical and biochemical markers, including serum lactate dehydrogenase, serum sodium concentration, serum alkaline phosphatase, serum bicarbonate, Karnofsky performance status (PS) and stage of disease (Cerny *et al*, 1987). Prognosis for patients with SCLC is poor, even in those with early stage (non-metastatic) SCLC. From the time of diagnosis, the median ranges of survival for LD-SCLC and ED-SCLC are 15–20 and 8–13 months, respectively. Approximately 20–40% of patients with LD-SCLC and 5% of patients with ED-SCLC survive for 2 years (Lally *et al*, 2007).

## Treatment of limited stage

**Figure 1:**
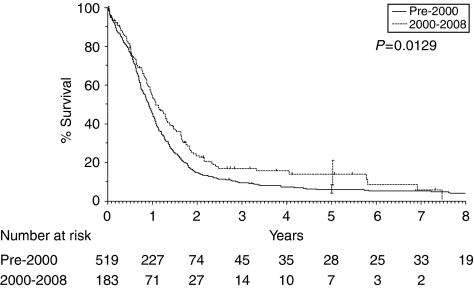
Survival rates of patients with LD-SCLC pre-2000: 5-year survival 13.9% vs 6.1% 2-year survival 17% vs 27%. (Unpublished data from the Royal Marsden Hospital, UK.)

The survival of patients with LD-SCLC has improved over the past 20 years, with a 5-year survival rate of 13.9% compared with 6% before 2000 in our own institution – the Royal Marsden Hospital (Figure 1). The positive outcome in LD seems to be multifactorial, with better staging, platinum-based chemotherapy and the use of prophylactic cranial irradiation (PCI) all exerting an effect. The addition of TRT to chemotherapy, both sequentially and, more recently, concomitantly, significantly reduces the risk of intrathoracic failures, resulting in improvements in long-term survival in this population (Pignon *et al*, 1992).

### Surgery

The function of surgery has never been fully developed because of the apparent failure of this treatment modality in two randomised trials reported in 1976 and 1994. Both trials did not show any survival advantage for surgery alone or in combination with TRT, compared with radiotherapy alone (Fox and Scadding, 1973; Lad *et al*, 1994). A recent review of these data suggests that the ‘intention to treat’ results may have misrepresented the effect of surgery, as not all patients randomised to surgery and included in the analyses actually underwent a complete resection. In fact, only 48% and 77% of patients, respectively, did so, meaning that the impact of surgery may have been underestimated.

A more recent retrospective analysis of surgery for patients with stage I–IIIB at the Royal Brompton Hospital reported a 5-year progression-free survival (PFS) of 46% (Lim and Goldstraw, 2008). Other published prospective series also suggest that surgery, after induction chemotherapy, can achieve high local control rates in early stage SCLC (stage I–III) with favourable long-term survival results (Fujimori *et al*, 1997; Eberhardt *et al*, 1999). The survival rates reported are higher than the historical control, with 63% and 46% of patients alive at 3 and 5 years, respectively. To validate this hypothesis, a number of randomised trials have recently been designed to compare surgery, in combination with chemotherapy or chemoradiotherapy, with the current standard of treatment with chemoradiotherapy using modern staging methods.

### Chemoradiation

Combined modality therapy with chemotherapy and thoracic irradiation is the current accepted standard for patients with LD-SCLC (NCCN, 2008). Two meta-analyses have established the function of TRT in the management of patients with LD (Pignon *et al*, 1992; Warde and Payne, 1992). Both reports found an improved intrathoracic tumour control rate with combined modality patients and statistically improved survival with an absolute benefit in overall survival (OS) at 3 years (Pignon *et al*, 1992) and 2 years (Warde and Payne, 1992) of 5.4%. Treatment-related toxicity is worse with combination treatments, but is acceptable in patients with a good PS.

There are a number of unresolved issues in the delivery of chemotherapy and radical radiotherapy in LD-SCLC including timing of TRT, early *vs* late, concomitant or sequential, total dose and fractionation schedule. Many trials have addressed the timing issues, but they have differed in trial design as well as the chemotherapy regimen used as reviewed recently by Socinski and Stinchcombe (2007).

A number of meta-analyses have shown a modest survival benefit with early compared with delayed concurrent TRT, most notably in combination with a cisplatin-based regimen (Fried *et al*, 2004; Huncharek and McGarry, 2004; Socinski and Stinchcombe, 2007). Additional analyses indicate that the overall treatment time for TRT, as well as the time of TRT initiation, influences survival. A Cochrane review of seven trials observed a significant survival benefit at 5 years for patients who had early radiotherapy delivered within an overall treatment time of 30 days (Pijls-Johannesma *et al*, 2005). A second analysis found that patients who received their last dose of TRT <30 days after the first dose of chemotherapy had a significantly greater likelihood of survival at 5 years compared with completion of TRT >30 days after chemotherapy initiation (De Ruysscher *et al*, 2006).

A landmark study by Turrisi *et al* (1999) showed that a hyperfractionation regimen of 45 Gy given twice daily over a 3-week period was associated with significantly improved OS at 5 years compared with 45 Gy once daily over 5 weeks (26% *vs* 16% *P*=0.04). Tolerability was comparable between the groups, although osophagitis was reported significantly more frequently in the twice-daily dosing group. Relapse rates in patients given once-daily TRT were significantly higher leading to the suggestion that patients in this arm were under-dosed. A trial has recently commenced comparing 45 Gy twice daily in 1.5 Gy fractions *vs* 66 Gy once daily in 2 Gy fractions that should provide further guidance on optimisation and standardisation of radiotherapy for LD-SCLC (Faivre-Finn and Falk, 2009).

It should be noted that EP remains the only chemotherapy that can be delivered at full dose in combination with radiotherapy in LD-SCLC.

## Treatment of ED

Chemotherapy is the main treatment for ED-SCLC. Treatments in the past including oral etoposide, standard- and high-dose regimens have been extensively reviewed by the authors in an earlier publication (Popat and O’Brien, 2005). Combination therapy with EP is considered the standard first-line regimen (Sundstrom *et al*, 2002), but a randomised Phase III trial failed to prove a definitive survival benefit for EP compared with the cyclophosphamide, doxorubicin and vincristine regimen (CAV) (Roth *et al*, 1992). In a recent trial (Sundstrom *et al*, 2002), a significant survival advantage in favour of the EP arm was seen for LD patients, but only a trend in survival benefit was seen in the ED group, with a median survival of 8.4 months as compared with 6.5 months in the CAV arm (*P*=0.21). The recently published Cochrane review in SCLC concluded that there was no statistical survival benefit from platinum-based regimens. There was an improvement in complete response rates, which is important in LD; however, there was greater toxicity in terms of emesis, anaemia and thrombocytopaenia. LD-SCLC and ED-SCLC were not separated. The review highlights the lack of quality-of-life (QoL) data. Major differences in QoL between an anthracycline and platinum-based regimen are not expected with modern powerful anti-emetics, and the haematological toxicities of anaemia and thrombocytopaenia can be managed (Amarasena *et al*, 2008). It is unlikely that a head-to-head study will be repeated with QoL assessment at this point in time.

A randomised Phase III study of EP *vs* carboplatin and etoposide (CE) showed no differences in response and survival between the two treatment arms, although the study was not powered for non-inferiority (Skarlos *et al*, 1994). A more recent randomised trial comparing split doses of EP *vs* CE in elderly or poor-risk patients with ED-SCLC also failed to show any significant difference in response rate or survival (Okamoto *et al*, 2007). Given these data, several cytotoxic drugs identified as active against SCLC have been tested in combination with cisplatin or carboplatin (Table 1).

A study conducted by the Japan Clinical Oncology Group showed a significant survival benefit for irinotecan and cisplatin combination *vs* EP (Noda *et al*, 2002), but a second study in US patients of the same agents failed to show any significant difference (Hanna *et al*, 2006a). A recent pharmacogenomic analysis of Japanese and US patients treated with carboplatin and paclitaxel for non-SCLC revealed that differences in outcomes for survival and toxicity could be explained by genotypic variations between the two nationalities (Gandara *et al*, 2009). The investigators found significant differences between Japanese and US patients in the frequency of variant alleles for genes encoding enzymes involved in paclitaxel metabolism and DNA repair. Exploratory analyses revealed significant associations between some of these variant alleles and patients’ treatment response and survival. A similar genetic basis may explain the different responses to irinotecan and cisplatin in SCLC. Indeed, it is established that the variety in allelic distribution of enzymes involved in irinotecan metabolism differ between Japanese and Caucasian patients (Ando *et al*, 2002).

Topotecan has shown significant anti-tumour activity and symptom palliation in relapsed SCLC. The doublet oral topotecan plus cisplatin (TC) showed both similar benefit and toxicity to EP. Grades 3 and 4 neutropaenia were more frequent with EP (84% *vs* 59%), whereas TC caused a higher rate of anaemia (38% *vs* 21%), thrombocytopaenia (38% *vs* 23%) and diarrhoea (33% *vs* 18%), but a lower rate of alopecia (TC 24% *vs* PE 40%) (Eckardt *et al*, 2006; Heigener *et al*, 2008).

Two Phase III randomised studies have investigated the possible function of paclitaxel in SCLC by adding paclitaxel to the platinum/etoposide doublet. Both trials led to similar conclusions and failed to show a benefit in survival for the experimental arm, but reported an increase in haematological and non-haematological toxicity, and a higher rate of toxicity-related death (Mavroudis *et al*, 2001; Niell *et al*, 2005).

A Phase III trial, evaluating pemetrexed and carboplatin, which was closed after a planned interim analysis showed inferior PFS in the experimental arm compared with the standard etoposide and platinum combination (Socinski *et al*, 2009).

A non-inferiority trial was designed to determine whether gemcitabine plus carboplatin (GC) would be less toxic, and associated with better QoL when compared with EP chemotherapy. The GC chemotherapy achieved survival rate, response rate and time to progression equivalent to EP. The two regimens had a different toxicity profile; a higher rate of grades 2–3 nausea and alopecia occurred in EP-treated patients, whereas more frequent grades 3 and 4 haematological toxicity were seen with the GC schedule (Lee *et al*, 2009). A more realistic comparison would have been GC and CE, and a comparative study of these combinations would be of interest. The GC regimen is useful, particularly in patients with mixed small cell and non-small cell tumours, and for those for whom alopecia is a real problem.

To summarise these results, all the recent randomised trials using later generation cytotoxics have not had a significant impact on standard care for SCLC, failing to identify a new platinum-based combination over the established platinum etoposide.

## New agents and ongoing research

Amrubicin is a synthetic anthracycline and a potent topoisomerase II inhibitor approved in Japan for the treatment of SCLC. As a first-line therapy for ED, amrubicin in combination with cisplatin achieved an impressive response rate of 88% and a median OS of 13.6 months in Japan (Ohe *et al*, 2005). Clinical trials are ongoing in the United States and Europe to determine whether amrubicin will be effective in other ethnic groups.

Targeted agents are also an area of considerable interest given their success in other tumour types. The SCLC has been identified as a highly angiogenic tumour and a number of pro-angiogenic circulating factors have been implicated in this disease. Thalidomide has anti-angiogenic properties and has been investigated earlier in combination with first-line chemotherapy and as maintenance therapy in Phase II trials (Dowlati *et al*, 2007; Lee *et al*, 2008). However, two large randomised Phase III trials have failed to show any benefit for thalidomide plus chemotherapy over chemotherapy alone (Pujol *et al*, 2007; Lee *et al*, 2009). In the most recent studies, an analysis of data from over 700 patients failed to show any difference in OS or PFS between the groups, but did show a significant increase in the risk of thrombotic events with thalidomide (Lee *et al*, 2009).

Maintenance therapy with vandetanib (ZD6474), a VEGF receptor tyrosine kinase inhibitor, did not increase OS or PFS when compared with placebo (Arnold *et al*, 2007). In addition, bevacizumab in first-line treatment of SCLC – in combination with either cisplatin and etoposide or cisplatin and irinotecan – has not yielded results as promising as was expected (Ready *et al*, 2007; Sandler *et al*, 2007); however, further results are awaited. Taken together, these data suggest that targeting angiogenesis in SCLC with current approaches may not work as well as in other tumour types.

Increased understanding of the molecular mechanism of tumour resistance to apoptosis has led to the development of promising targeted therapies, which selectively modulate pro- and anti-apoptotic proteins. Small molecule inhibitors of *bcl-2* family anti-apoptotic members are currently under evaluation in Phase I and II trials. The first Phase II trial with the *bcl-2* anti-sense oligonucleotide oblimersen as initial therapy for ED-SCLC has been negative, suggesting worse outcome for patients receiving oblimersen in association with CE, compared with CE alone. The response rates were the same in both treatment arms (61% *vs* 60%), whereas the percentage of patients alive at 1 year was 24% with oblimersen and 47% without oblimersen (Rudin *et al*, 2008).

To date, none of the new-targeted agents investigated have been found to alter the clinical history of SCLC.

### Prophylactic cranial irradiation

Approximately 14–24% of patients with SCLC have detectable brain metastases (BM) at the time of initial diagnosis (Argiris and Murren, 2001). Data regarding the efficacy of chemotherapy in patients with brain relapse is scanty, although responses do occur (Postmus *et al*, 1989, 1995; Groen *et al*, 1993). Survival after metastasis to the brain is short, ranging from about 3 to 5 months (Postmus *et al*, 1998, 2000).

Trials conducted in the late 1980s and early 1990s, and two subsequent meta-analyses, have confirmed that PCI significantly improves survival for patients with SCLC who achieved a complete response (using chest X-rays) after induction treatment, with an absolute improvement in OS of 5.4% at 3 years (Auperin *et al*, 1999; Prophylactic-Cranial-Irradiation-Overview-Collaborative-Group, 2000). As revealed by a CT scan, PCI is also now offered to patients with a good partial response.

The function of PCI in patients with ED-SCLC after chemotherapy has recently been investigated in a randomised Phase III trial conducted by the EORTC lung group (Slotman *et al*, 2007). The incidence of symptomatic BM, the primary objective of the trial, was significantly reduced after PCI in patients who did not progress on initial systemic therapy. The cumulative risk of metastases at 1 year was 14.6% in the PCI arm compared with 40.4% in the control arm. The median survival increased from 5.4 to 6.7 months after randomisation, and the 1-year survival rate was 27.1% in the irradiation group and 13.3% in the control group. However, it should be noted that brain imaging was not conducted in this trial before randomisation unless patients displayed symptoms indicative of BM. Nevertheless, a recent UK survey reports quick implementation of PCI in patients with ED-SCLC in radiotherapy centres based on the EORTC data (Bayman *et al*, 2008, 2009).

## Treatment options for relapsed SCLC

Despite high initial response rates to chemotherapy, SCLC usually recurs within 1 year after treatment (Ardizzoni, 2004). It is estimated that 80% of patients with LD, and almost all patients with ED, relapse or experience disease progression (Clark and Ihde, 1998). As the prognosis is poor, symptom palliation and maintenance of QoL are important therapeutic goals in the relapsed setting (Gralla, 2004; Nicum and O’Brien, 2007).

### Chemotherapy options

On the basis of retrospective data, distinction has been made between (1) sensitive patients, that is those with a response to first-line therapy and a treatment-free interval of at least 90 days, (2) resistant patients, that is relapse within 90 days and (3) refractory patients, that is no response to first-line treatment (Vincent *et al*, 1988; Fischer and Arcaro, 2008). The PS and sensitivity to initial chemotherapy were found to be prognostic and predictive variables for chemotherapy outcome in recurrent SCLC patients (Kim *et al*, 2008). The relevance of these categories has recently been called into question with respect to re-treatment decisions (O’Brien *et al*, 2006).

The CAV may be used after first-line treatment with EP, with a response rate of 8–28% (von Pawel *et al*, 1999). A trial of the Norwegian lung cancer study group has evaluated the benefit of crossover chemotherapy with CAV at relapse after primary treatment with EP, compared with EP at relapse after CAV. There was no survival difference between the two crossover treatment groups (median survival after relapse was 3.9 and 4.5 months, respectively), and no differences in outcomes were observed in either resistant or sensitive patients. Nevertheless, these data are from a selected subgroup of patients, because only a limited number of patients (42%) from the initial-treated population were considered to be suitable for second-line chemotherapy at the time of disease progression. Moreover, a comparison between the second-line chemotherapy and the best supportive care group, in a non-randomised design, revealed a significantly better survival rate in favour of the chemotherapy group, although this difference could be explained by more negative prognostic factors in the best supportive care group (Sundstrom *et al*, 2005).

Single-agent topotecan is currently the only approved drug for the treatment of patients with SCLC who have failed or relapsed after first-line chemotherapy. Topotecan is available in both intravenous and oral formulations and randomised studies have suggested that both have similar clinical activity in SCLC (von Pawel *et al*, 2001; Eckardt, 2003). A randomised Phase III trial has shown significant benefit with oral topotecan plus best supportive care *vs* best supportive care only in relapsed patients unsuitable for intravenous regimens (Table 2). The OS was significantly longer in the topotecan group (median survival 25.9 *vs* 13.9 weeks), and survival was preserved in patients with a short time to progression after first-line therapy (60 days) and in those with a PS score of 2. Moreover, patient QoL and symptom control was significantly greater in patients who received topotecan (O’Brien *et al*, 2006). A randomised trial comparing intravenous topotecan with the CAV regimen observed comparable response rates (24.3% and 18.3%, respectively) and median survival (25.0 and 24.7 weeks), although topotecan was associated with greater symptom improvement in terms of improved dyspnoea, anorexia, fatigue, insomnia and daily activity (von Pawel *et al*, 1999).

### Other evaluated drugs

Over the past years, several cytotoxic agents, including taxanes, gemcitabine, vinorelbine and irinotecan, have been investigated for second-line treatment, as either single agent or combination (Table 3). Out of the many Phase II trials, with a relatively small number of patients and uneven distribution of sensitive *vs* refractory disease, some agents, such as paclitaxel, irinotecan and temozolamide, have shown some degree of activity. Nevertheless, the lack of comparative trials prevents any formal conclusions.

Pemetrexed (Table 4), recently tested in three Phase II studies, has shown minimal activity in relapsed SCLC patients. High-dose pemetrexed can be given without significant increase in serious toxicities, but this does not seem to increase efficacy (Hanna *et al*, 2006b; Gronberg *et al*, 2008; Socinski *et al*, 2008).

### New agents and ongoing research

As well as in first-line chemotherapy, amrubicin have shown impressive results for the second-line treatment of relapsed SCLC. High response rates (between 37% and 60%) have been reported for single-agent amrubicin in three Phase II Japanese studies conducted in a population with sensitive and resistant relapses (Kato *et al*, 2006; Kudoh *et al*, 2006; Onoda *et al*, 2006). The overall median survival was 11.2 and 8.8 months for sensitive and resistant patients, respectively (Kato *et al*, 2006; Onoda *et al*, 2006). Interestingly, the response rate and the median survival time were similar in both sensitive and resistant patients (Table 4). A US Phase II trial has investigated single-agent amrubicin in patients with refractory or resistant SCLC (Ettinger *et al*, 2008). Activity was observed and the most frequent toxicity was myelosuppression, but no classic anthracycline-induced cardiotoxicity has been observed.

A randomised Phase II trial has compared amrubicin and topotecan in earlier treated SCLC. This study further supports the efficacy of amrubicin in both sensitive (overall response rate of 53%) and resistant patients (overall response rate of 17%). A higher response rate was achieved with amrubicin compared with topotecan (Inoue *et al*, 2008). This Japanese study unfortunately used topotecan at a dose of 1 mg m^–2^ rather than the current recommended dose of 1.5 mg m^–2^. Preliminary results of a second Phase II trial seem to confirm a trend in favour of amrubicin over topotecan as single-agent chemotherapy for sensitive relapsed SCLC in terms of response rate, but PFS was not statistically different. Further evaluations are currently ongoing within a Phase III setting.

At present, a second drug is under investigation in a Phase III setting. Picoplatin is a platinum analogue with some activity in relapsed SCLC, as shown in an earlier Phase II trial conducted in refractory, resistant and sensitive patients (Eckardt *et al*, 2009). The Phase III study evaluates picoplatin plus best supportive care *vs* best supportive care alone in both refractory and relapsed patients (SPEAR trial).

Targeted therapy is currently being explored in relapsed SCLC. Some of the first agents investigated as second-line therapy were imatinib and gefitinib; however, both agents were unsuccessful (Table 3). Current research is now focused on angiogenesis inhibitors (Table 3).

From preliminary results of a Phase II trial, bevacizumab in combination with paclitaxel in chemosensitive relapsed SCLC seems to be an active regimen (Jalal *et al*, 2008). Cediranib, a highly potent inhibitor of VEGFR-1, -2 and -3, has also shown limited anti-cancer activity as monotherapy in chemosensitive relapsed patients (Ramalingam *et al*, 2008).

Sorafenib and sunitinib are multikinase inhibitors acting on pathways involved in tumour progression and angiogenesis, and are both undergoing investigation for the treatment of SCLC in either the first- or second-line setting. The only data available so far are on sorafenib, which seems to be a promising agent with a median survival of 7 and 5 months in platinum-sensitive and platinum-refractory patients, respectively (Gitlitz *et al*, 2008). This compared favourably with historical controls receiving salvage chemotherapy. A Phase I trial of weekly topotecan in combination with sorafenib in treatment of relapsed SCLC has been commenced.

NKH-1 (or CD56) is a neural adhesion molecule that is highly expressed in SCLC (Fischer and Arcaro, 2008). The immunotoxin N901-bR is a murine monoclonal antibody directed against CD56. Hu 901-DM1 is an immunoconjugate created by the conjugation of the maytansinoid drug DM 1 to a humanised version of the murine monoclonal antibody N901. At a dose of 60 mg m^–2^ per week, 2 out of 10 patients with relapsed SCLC obtained a partial response and another one a minor response; this study is ongoing (Fossella *et al*, 2005).

### Proteosome inhibitors

Bortezomib targets the ubiquitin–proteosome pathway interfering with p21, p27, p53, cyclins D, E and A, nuclear factor *κ*B and members of the Bax family. In a Phase II trial using bortezomib at a dose of 1.3 mg m^–2^ on Days 1, 4, 8 and 11 every 21 days, only one partial response was obtained. Further studies are, therefore, underway to assess the benefit of combining bortezomib with topotecan in the second-line setting (Johl *et al*, 2005).

## Conclusion

The SCLC is a significant healthcare problem worldwide because of its aggressive nature and high propensity for relapse. Combination chemotherapy continues to be the mainstay of SCLC treatment in the first-line setting; the combination of cisplatin or carboplatin with etoposide is the most commonly used regimen. Despite relatively high initial response rates, most patients with SCLC eventually relapse, but treatment options for patients with relapsed SCLC are limited. Topotecan is currently the only approved single-agent for second-line therapy and recent data with oral topotecan show that more of these patients than suggested earlier can achieve further responses, symptom control and survival prolongation.

A number of other chemotherapeutic agents, including irinotecan, paclitaxel and amrubicin, have shown some activity in small Phase II/III trials when used as monotherapy or in combination with other cytotoxic agents. Recent progress in the understanding of the biology of SCLC has led to the identification of crucial signalling pathways and the subsequent development of targeted therapies. Several novel molecules are presently undergoing evaluation, and represent potential future additions to the treatment repertoire for SCLC.

## Conflict of interest

This article was supported by GlaxoSmithKline. M O’Brien has performed paid lectures and consultancies for GlaxoSmithKline and Celgene.

## Figures and Tables

**Table 1:** Phase II/III studies of combination chemotherapy in patients with ED-SCLC

			**Experimental regimen**		**Standard regimen**	**Outcomes**		
**Author**	**Staging**	**Patients (*n*)**		**Schedule**	**Schedule**	**Overall survival (months)**		**RR**
Noda *et al* (2002)	ED	154	Irinotecan/cisplatin	I: 60 mg /m^–2^ days 1, 8, 15 P: 60 mg m^–2^ day 1; q4w	E: 100 mg m^–2^ days 1, 2, 3 P: 80 mg m^–2^ day 1; q3w	12.8 *vs* 9.4 months	+ve	+ve
Hanna *et al* (2006a)	ED	331		I: 65 mg m^–2^ days 1, 8 P: 30 mg m^–2^ days 1, 8; q3w	E: 120 mg m^–2^ days 1,2,3 P: 60 mg m^–2^ day 1; q3w	9.3 *vs* 10.2 months	NS	NS
Lara *et al* (2009)	ED	645		I: 60 mg m^–2^ days 1, 8, 15 P: 60 mg m^–2^ day 1; q4w	E: 100 mg m^–2^ days 1,2,3 P: 80 mg m^–2^ day 1; q3w	9.9 *vs* 9.1 months	NS	NS
Hermes *et al* (2008)	ED	220	Irinotecan/carboplatin	I: 175 mg m^–2^ day 1 Ca: AUC 4 day 1; q3w	E: 120 mg m^–2^ orally days 1–5 Ca: AUC 4 day 1; q3w	8.5 *vs* 7.1 months	+ve	/
Socinski *et al* (2009)	ED	733	Pemetrexed/carboplatin	Pe: 500 mg m^–2^ day 1 Ca: AUC 5 day 1; q3w	E: 100 mg m^–2^ days 1, 2, 3 Ca: AUC 5 day 1; q3w	7.29 *vs* 9.56 months	–ve	–ve
Niell *et al* (2005)	ED	587	Paclitaxel/etoposide/cisplatin	Pa: 175 mg m^–2^ day 1 E: 80 mg m^–2^ days 1–3 P: 80 mg m^–2^ day 1; q3w	E: 80 mg m^–2^ days 1, 2, 3 P: 80 mg m^–2^ day 1; q3w	10.6 *vs* 9.9 months	NS	+ve
Mavroudis *et al* (2001)	ED, LD	133		Pa: 175 mg m^–2^ day 1 E: 80 mg m^–2^ days 1–4 P: 80 mg m^–2^ day 2; q3w	E: 120 mg m^–2^ days 1, 2, 3 P: 80 mg m^–2^ day 1; q3w	9.5 *vs* 10.5 months	NS	NS
Heigener *et al* (2008)	ED	795	Topotecan/cisplatin	T: 1 mg m^–2^ days 1–5 P: 75 mg m^–2^ day 1; q3w	E: 100 mg m^–2^ days 1, 2, 3 P: 75 mg m^–2^ day 1; q3w	10.3 *vs* 9.4 months	Non-inferior	+ve
Eckardt *et al* (2006)	ED	784		T: 1.7 mg m^–2^ oral days 1–5 P: 60 mg m^–2^ day 5; q3w	E: 100 mg m^–2^ days 1, 2, 3 P: 80 mg m^–2^ day 1; q 3w	39.3 *vs* 40.3 weeks	Non-inferior	NS
Lee *et al* (2009)	ED, LD, poor prognoses	241	Gemcitabine/carboplatin	G: 1200 mg m^–2^ days 1, 8 Ca: AUC 5 day 1; q3w	E: 120 mg m^–2^ day 1; 100 mg m^–2^ bd orally days 2, 3 P: 60 mg m^–2^ day 1; q3w	8 *vs* 8.1 months	Non-inferior	NS

Abbreviations: P=cisplatin; Ca=carboplatin; E=etoposide; I=irinotecan; Pe=pemetrexed; Pa=paclitaxel; T=topotecan; G=gemcitabine; RR=response rate; NS=not significant; AUC=area under the curve; ED=extensive disease; LD=limited disease; SCLC=small cell lung cancer.

**Table 2:** Randomised trials for the treatment of relapsed SCLC

					**Results**					
					**Response rate (%)**					
**Authors**	**Study type**	**Regimen**	**Schedule**	**Patients (*n*)**	**ORR**	**CR**	**PR**	**SD**	**Median TTP (weeks)**	**Median survival (weeks)**
von Pawel *et al* (1999)	Randomised Phase II	CAV *vs* topotecan	Cyclophosphamide 1000 mg m^–2^ + doxorubicin 45 mg m^–2^ + vincristine 2 mg m^–2^, day 1, q3w	104	18.3	1	18	12	—	24.7
			IV topotecan: 1.5 mg m^–2^, days 1–5, q3w	107	24.3	0	26	21	—	25.0
von Pawel *et al* (2001)	Randomised Phase II	Oral topotecan *vs* IV topotecan	Oral topotecan: 2.3 mg m^–2^, days 1–5, q3w	52	23	2	21	19	14.9	32
			IV topotecan: 1.5 mg m^–2^, days 1–5, q3w	54	15	4	11	30	13.1	25
Eckardt (2007)	Randomised Phase III	Oral topotecan *vs* IV topotecan	Oral topotecan: 2.3 mg m^–2^, days 1–5, q3w	153	18	—	—	18	—	33
			IV topotecan: 1.5 mg m^–2^_,_ days 1–5, q3w	151	22	—	—	23	—	35
O’Brien *et al* (2006)	Randomised Phase III	Oral topotecan + BSC *vs* BSC alone	Oral topotecan: 2.3 mg m^–2^, days 1–5, q3w	71	7	0	7	44	16.3	25.9
			BSC alone	70	—	—	—	—	—	13.9

Abbreviations: ORR=overall response rate; CR=complete response; PR=partial response; SD=stable disease; TTP=time to progression; CAV=cyclophosphamide, doxorubicin and vincristine; BSC=best supportive care; SCLC=small cell ling cancer.

**Table 3:** Clinical studies of targeted agents for the treatment of SCLC

						**Result**		
**Authors**	**Targeted agent**	**Combination regimen**	**Targeted population**	**Phase**	**Sample size**	**RR**	**OS/PFS (months)**	**Conclusion**
*TKs inhibitors*								
Dy *et al* (2005)	Imatinib	—	Relapsed, resistant/sensitive [c-Kit +]	II	29	No PR No SD	OS: R=3.9 S=5.3	No clinical activity
Krug *et al* (2005)		—	Relapsed, resistant/sensitive [c-Kit +]	II	12	No PR No SD	OS: 2	No clinical activity
Johnson *et al* (2003)		—	ES, untreated relapsed, sensitive	II	19	No PR	—	No clinical activity
Schneider *et al* (2006)		—	ES, if no PD after I-line IP × 4 [c-Kit +]	II	14	No PR	OS: 10	Disease stability not maintained
Spigel *et al* (2007)		Carboplatin/irinotecan	ES, untreated	II	69	PR 66%	OS: 8.4	No improvement in results from chemotherapy alone
Moore *et al* (2006)	Gefitinib	—	Relapsed, resistant/sensitive	II	19	No PR 10% SD	OS: 206 days	No clinical activity
								
*Anti-angiogenic agents*								
Sandler *et al* (2007)	Bevacizumab	Cisplatin/etoposide	ES, untreated	II	64	OR 69%	PFS at 6 months: 33%	Promising results
Ready *et al* (2007)		Irinotecan/cisplatin	ES, untreated	II	72	CR 3% PR 71%	OS: 11.7	Primary end point not reached
Jalal *et al* (2008)		Paclitaxel	Relapsed, sensitive	II	34	PR 11% SD 55%	OS: 21 weeks	Active regimen
Pujol *et al* (2007)	Thalidomide	PCDE	ES, after response to PCDE × 2	III	119	—	OS: 11.7/8.7 (NS)	Thalidomide did not improve survival
Lee *et al* (2008)		Carboplatin/etoposide	ES and LS, untreated	III	724	—	OS: 10.2/10.5 (NS)	Thalidomide did not improve survival
Gitlitz *et al* (2008)	Sorafenib	**—**	Relapsed (platinum-treated)	II	81	PR 4% SD 32%	−7, S −5, R	Clinical activity
Ramalingam *et al* (2008)	Cediranib (ZD2171)	**—**	Relapsed (platinum-treated)	II	25	PR: 1 patient SD: 9 patients	PFS 1.2	No clinical activity
Arnold *et al* (2007)	Vandetanib (ZD6474)	**—**	ES and LS, untreated. If no PD after first-line platinum-based (and PCI/TRT)	II	107	—	PFS and OS NS	No efficacy as maintenance therapy

Abbreviations: RR=response rate; PR=partial response; SD=stable disease; OS=overall survival; PD=progressive disease; ES=early-stage; PFS=progression-free survival; CR=complete response; PCDE=etoposide plus cisplatin and cyclophosphamide plus 4′-epidoxorubicin; LS=late stage; S=sensitive; R=resistant; NS=not significant; PCI=prophylactic cranial irradiation; TRT=thoracic radiotherapy; OR=overall response.

**Table 4:** Evaluated drugs in relapsed SCLC

						**Results**		
**Drug**	**Dose/schedule**	**Authors**	**Population**	**Phase**	**Patients (*n*)**	**Response (%)**	**OS**	**Conclusion**
Gemcitabine	1250 mg m^–2^ days 1, 8; q3w	Hoang *et al* (2003)	Se, Rs, Re	II	27	No response	6.4 months	Limited activity
	1000 mg m^–2^ days 1, 8, 15; q4w	van der Lee *et al* (2001)	Rs, Re (76% >1 earlier line)	II	41	13%	17 weeks	Modest activity
	1000 mg m^–2^ days 1, 8, 15 q4w	Masters *et al* (2003)	Se, Rs, Re	II	46	11.9%	7.1 months	Modest activity
Irinotecan	100 mg m^–2^ weekly	Masuda *et al* (1992)	Se, Rs, Re	II	16	47%	6.8 months	Active agent
Paclitaxel	175 mg m^–2^; q3w	Smit *et al* (1998)	Rs	II	24	29%	100 days	Active agent
	200 mg m^–2^; q3w	Joos *et al* (2004)	Rs, Re	II	44	20%	4 months	Active agent
Vinorelbine	25 mg m^–2^ weekly	Furuse *et al* (1996)	Se, Rs, Re	II	24	12.5%	—	Modest activity
	30 mg m^–2^ weekly	Jassem *et al* (1993)	Se	II	26	16%	—	Modest activity
Pemetrexed	500 mg m^–2^; q3w	Hanna *et al* (2006b)	Se, Rs	II	43	Se: 1 PR Rs: 1 PR	—	Minimal activity
	900 mg m^–2^; q3w	Gronberg *et al* (2008)	Se, Rs	II	34	Se: 4.5% Rs: 2.9%	17.6 weeks	Limited activity
	900 mg m^–2^; q3w	Socinski *et al* (2008)	Se, Rs	II	121	0.9% (1 PR in Se)	2.5–6.1 months	Minimal activity
Amrubicin	40 mg m^–2^ days 1–3; q3w	Onoda *et al* (2006)	Se, Rs	II	60	Se OR: 52% Rs OR: 50%	Se: 11.6 months Rs: 10.3 months	Significant activity
	45 mg m^–2^ days 1–3; q3w	Kato *et al* (2006)	Se, Rs	II	35	Se OR: 50% Rs OR: 60%	8.8 months	Significant activity
	40 mg m^–2^ days 1–3; q3w	Kudoh *et al* (2006)	Se, Rs	II	19	OR: 37%	—	Active agent
	40 mg m^–2^ days 1–3; q3w	Ettinger *et al* (2008)	Rs, Re	II	63	PR: 13/39	—	Active agent
	Amrubicin: 40 mg m^–2^ days 1–3; q3w Topotecan: 1 mg m^–2^ days 1–5	Inoue *et al* (2008)	Se, Rs	II	60	38% *vs* 13%	—	Amrubicin may be superior to topotecan
Picoplatin	150 mg m^–2^; q3w	Bentzion *et al* (2007)	Se, Rs, Re	II	77	—	28.1 weeks	Compares favourably with other therapeutic options

Abbreviations: OS=overall survival; Se=sensitive (initially responded and then relapsed/progressed between 60 and 180 days); Rs=resistant (initially responded to first-line platinum-containing chemotherapy and then relapsed/progressed within 60–90 days); Re=refractory (failed or progressed with first-line platinum-containing chemotherapy); PR=partial response; OR=overall response.
